# The Thioredoxin TRX-1 Modulates the Function of the Insulin-Like Neuropeptide DAF-28 during Dauer Formation in *Caenorhabditis elegans*

**DOI:** 10.1371/journal.pone.0016561

**Published:** 2011-01-27

**Authors:** Juan Carlos Fierro-González, Astrid Cornils, Joy Alcedo, Antonio Miranda-Vizuete, Peter Swoboda

**Affiliations:** 1 Department of Biosciences and Nutrition, Center for Biosciences at NOVUM, Karolinska Institute, Huddinge, Sweden; 2 Friedrich Miescher Institute for Biomedical Research, Basel, Switzerland; 3 Departamento de Fisiología, Anatomía y Biología Celular, Centro Andaluz de Biología del Desarrollo - Consejo Superior de Investigaciones Científicas (CABD-CSIC), Universidad Pablo de Olavide, Sevilla, Spain; 4 Instituto de Biomedicina de Sevilla, Hospital Universitario Virgen del Rocío, Consejo Superior de Investigaciones Científicas, Universidad de Sevilla, Sevilla, Spain; University Medical Center Groningen, The Netherlands

## Abstract

Thioredoxins comprise a conserved family of redox regulators involved in many biological processes, including stress resistance and aging. We report that the *C. elegans* thioredoxin TRX-1 acts in ASJ head sensory neurons as a novel modulator of the insulin-like neuropeptide DAF-28 during dauer formation. We show that increased formation of stress-resistant, long-lived dauer larvae in mutants for the gene encoding the insulin-like neuropeptide DAF-28 requires TRX-1 acting in ASJ neurons, upstream of the insulin-like receptor DAF-2. Genetic rescue experiments demonstrate that redox-independent functions of TRX-1 specifically in ASJ neurons are needed for the dauer formation constitutive (Daf-c) phenotype of *daf-28* mutants. GFP reporters of *trx-1* and *daf-28* show opposing expression patterns in dauers (i.e. *trx-1* is up-regulated and *daf-28* is down-regulated), an effect that is not observed in growing L2/L3 larvae. In addition, functional TRX-1 is required for the down-regulation of a GFP reporter of *daf-28* during dauer formation, a process that is likely subject to DAF-28-mediated feedback regulation. Our findings demonstrate that TRX-1 modulates DAF-28 signaling by contributing to the down-regulation of *daf-28* expression during dauer formation. We propose that TRX-1 acts as a fluctuating neuronal signaling modulator within ASJ neurons to monitor the adjustment of neuropeptide expression, including insulin-like proteins, during dauer formation in response to adverse environmental conditions.

## Introduction

Thioredoxins comprise a conserved family of proteins characterized by the so-called thioredoxin fold and the highly conserved cysteine–glycine–proline–cysteine (CGPC) catalytic active site (reviewed in [1], [2]). The two cysteines in the active site of thioredoxin are required for the reversible reduction of disulfide bonds in many target proteins. The functions of thioredoxins are extensive and mostly depend on their disulfide oxidoreductase attributes; in general, they can act as electron donors for metabolic enzymes, as antioxidants, or as redox regulators of signaling molecules and transcription factors (reviewed in [1]).

In some cases, thioredoxins have been reported to execute their specific functions in a redox-independent manner (reviewed in [2], [3]). For instance, the regulation of phage T7 DNA polymerase activity by *E. coli* Trx1 [4], [5], the cytokine function of human truncated thioredoxin (Trx80) [6], or the regulation of apoptosis signaling kinase 1 (ASK1) by human Trx1 [7], among others, are all functions carried out independently of their oxidoreductase activity. More recently, thioredoxins have also been shown to promote folding of proteins independently of their redox activities (reviewed in [3]).

In the nematode *Caenorhabditis elegans*, the gene *trx-1* encodes a thioredoxin found to be expressed in the ASJ sensory neuron pair [8], [9]. These neurons are implicated in the regulation of aging, the response to stress conditions and the control of a state of developmental arrest called the dauer larva [10], [11], [12]. Previously, we and others have shown that *trx-1* deletion shortens lifespan and increases sensitivity to oxidative stress, though it itself does not affect dauer formation [8], [9]. These findings implicate TRX-1 in processes that regulate aging and stress resistance, which raises the question whether it is involved in mechanisms implicated in the regulation of dauer formation.

The *C. elegans* dauer larva has evolved as a tightly-regulated, long-lived and stress-resistant developmental stage only triggered when the animals encounter harsh environmental conditions [13]. However, under circumstances in which genes involved in dauer development become inactivated due to mutations, animals arrest as dauers even under favorable conditions (dauer formation constitutive or Daf-c phenotype) [14]. Among the known *daf-c* genes whose functions are linked to ASJ, four have previously been well characterized for their role in the dauer formation pathway: *daf-11* (abnormal *da*uer *f*ormation-*11*), *daf-28, tax-2* (abnormal chemo*tax*is-*2*) and *tax-4* (Figure S1; [12], [15], [16], [17], [18]). Moreover, mutations in these four *daf-c* genes have also been reported to extend lifespan [19], [20], [21], providing an additional link between dauer formation and aging. *daf-11* encodes a transmembrane guanylyl cyclase, while *tax-2* and *tax-4* encode subunits of a cyclic guanosine monophosphate (cGMP)-gated ion channel; these three genes are expressed in subsets of amphid sensory neurons, including ASI and ASJ [15], [22]. DAF-11 regulates dauer formation by modulating the levels of cGMP in these neurons, to which TAX-2/TAX-4 respond [22], which then regulate the expression of transforming growth factor-beta (TGF-beta) [23] and insulin-like neuropeptides [18], [24] (Figure S1). There are ∼40 insulin-like neuropeptides in *C. elegans* (www.wormbase.org), and several, including DAF-28, have been reported to be produced in ASJ, among other neurons [18], [24]. To gain insight into whether TRX-1 plays a role in dauer formation in *C. elegans*, we analyzed at the cellular and genetic level its interaction with *daf-c* genes co-expressed in ASJ neurons.

We have identified the *C. elegans* thioredoxin TRX-1 as a novel modulator of the insulin-like neuropeptide DAF-28 during dauer formation. We found that *trx-1* suppresses the Daf-c phenotype of all *daf-28* insulin-like mutant alleles tested and that this suppression requires a functional DAF-2 insulin-like receptor. Genetic rescue experiments demonstrated that redox-independent functions of transgenic TRX-1 provided specifically in ASJ neurons can restore the suppression exerted by *trx-1* deletion on the Daf-c phenotype of *daf-28* mutants. The suppression observed at the genetic level is also manifest at the cellular level specifically during dauer formation: GFP reporters of *trx-1* and *daf-28* display opposing expression patterns in dauers, which is in contrast to what is observed in growing L2/L3 larvae. Moreover, functional TRX-1 is required for the down-regulation of a GFP reporter of *daf-28* during dauer formation, a mechanism that is likely mediated by DAF-28-dependent feedback regulation. Our data suggest that TRX-1 contributes to the regulation of insulin-like neuropeptide expression, in particular DAF-28, during dauer formation in response to a changing environment.

## Results

### *trx-1* has a novel synthetic dauer formation constitutive (Daf-c) phenotype

**Figure 1:**
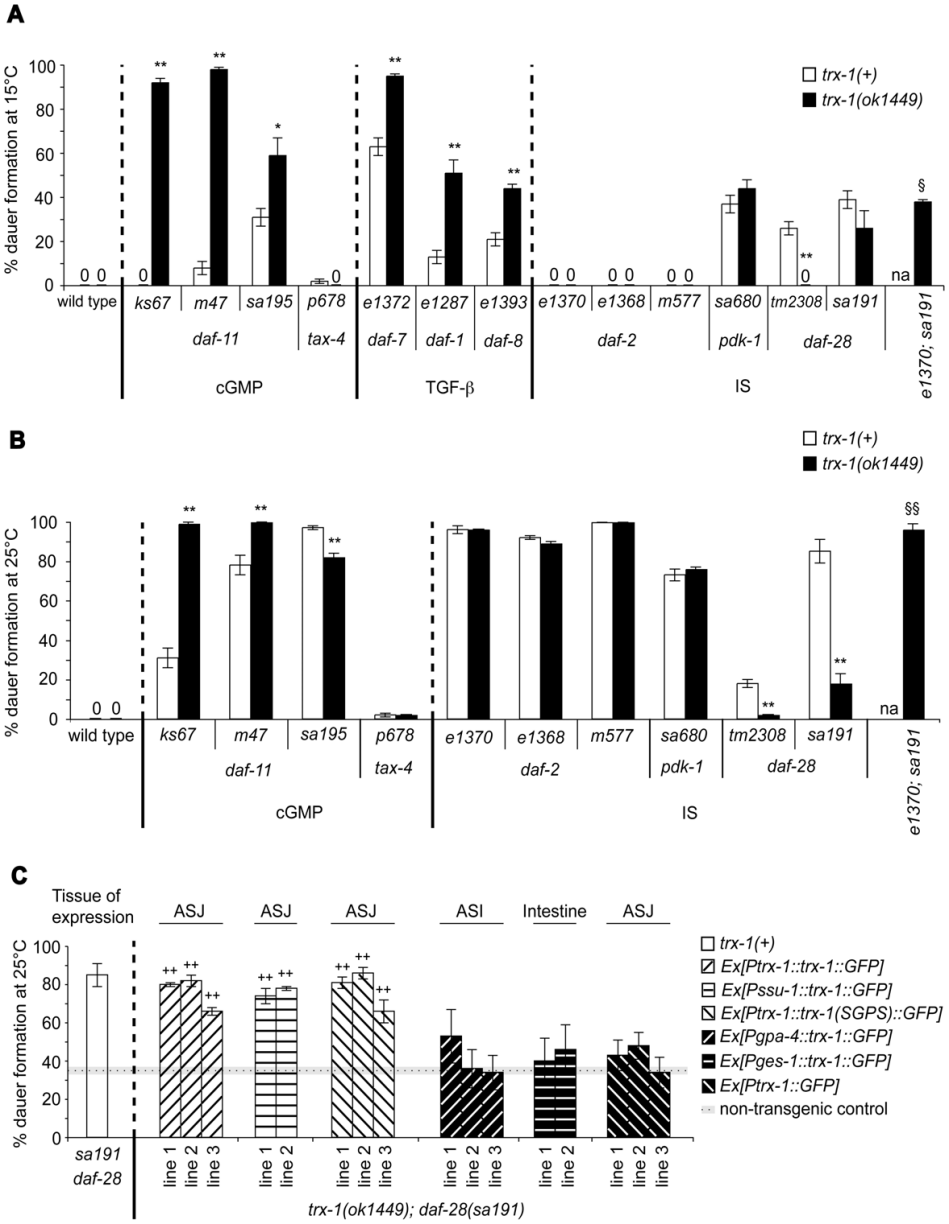
Redox-independent functions of TRX-1 in ASJ neurons modulate DAF-28 signaling during dauer formation. Dauer formation at 15°C (A) and 25°C (B) is shown for each of the *daf-c* mutant alleles tested in a *trx-1(+)* wild-type background (white bars) or in combination with a *trx-1(ok1449)* deletion null mutant allele (black bars). (C) Rescue of the suppression of the Daf-c phenotype in *trx-1(ok1449); daf-28(sa191)* transgenic animals (patterned bars) expressing either *Ptrx-1::trx-1::GFP*, *Pssu-1::trx-1::GFP*, *Ptrx-1::trx-1(SGPS)::GFP*, *Pgpa-4::trx-1::GFP*, *Pges-1::trx-1::GFP* or *Ptrx-1::GFP*. The cellular site of expression for each transgenic extrachromosomal array is outlined above the graph in panel C. The non-transgenic control represents the average of all assays performed with non-transgenic progeny that segregated from the same transgenic parents (dotted line) ± standard error of the mean (SEM, horizontal gray line). The white bar corresponding to the *daf-28(sa191)* single mutant in panel C, has been taken from panel B for comparison. In all three panels, each bar represents the average of 2–3 independent assays ± SEM, with more than 165 animals assayed in total per genotype. * *p*<0.05, ** *p*<0.01, ^§^ *p*<0.05 relative to *trx-1(ok1449); daf-28(sa191)*, ^§§^ *p*<0.01 relative to *trx-1(ok1449); daf-28(sa191)*, and ^++^ *p*<0.001 relative to non-transgenic animals, by chi-squared test (see also Tables 1, 2 and 3). na: not assayed. The molecular identities of all mutant alleles presented here are shown in Table S1.

To investigate whether *trx-1* plays a role in dauer formation, we first constructed double mutant combinations with different alleles of *daf-11*, since this gene is predicted to regulate dauer formation upstream of both the TGF-beta and insulin-like signaling (IS) pathways (Figure S1; [18], [23]). Previously, it has been suggested that ASJ neurons are required for the *daf-11* Daf-c phenotype, since laser ablation of ASJ suppresses the Daf-c phenotype of a *daf-11(sa195)* mutant [12]. We tested whether *trx-1* is also required for the *daf-11* Daf-c phenotype. The *trx-1(ok1449)* allele used in this study is a null mutation: a deletion in the coding region preventing translation of the protein (Table S1; [9]). *trx-1(ok1449)* enhanced the Daf-c phenotype of all *daf-11* alleles tested at 15°C (Figure 1A and Table 1). Even at 25°C, two of the three *daf-11* alleles tested showed a significant increase in dauer formation (Figure 1B and Table 2). Thus, *trx-1* has a novel synthetic Daf-c phenotype. In addition, the slight suppression of the *daf-11(sa195)* Daf-c phenotype exerted by *trx-1(ok1449)* at 25°C (Figure 1B and Table 2), partially phenocopies the effect of killing ASJ in a *daf-11(sa195)* single mutant background [12]. Together, our findings indicate that most of the Daf-c phenotype of *daf-11* mutants is TRX-1-independent, while a small fraction requires TRX-1. This dual effect of the *trx-1* mutation on *daf-11* for dauer formation is reminiscent, although not identical, to that reported for mutations in the cGMP-gated ion channel genes *tax-2* and *tax-4* [16]. *tax-2* and *tax-4* mutants show weak Daf-c phenotypes, which are epistatic to the strong *daf-11* Daf-c phenotypes [16]. *trx-1* deletion did not affect the weak Daf-c phenotype caused by *tax-4(p678)* (Figures 1A and 1B; Tables 1 and 2). Since mutations in *tax-4* essentially remove the input from *daf-11* signaling for dauer formation [16], *trx-1* deletion could not replicate the synthetic interaction observed with *daf-11* mutants. These results further support the notion of *tax-4* acting downstream of *daf-11* for dauer formation [16], with *trx-1* acting mostly independently of *daf-11* in the dauer formation pathway. We speculate that for the most part TRX-1 and DAF-11 are affecting a separate set of neurons for dauer formation (e.g. TRX-1 in ASJ and DAF-11 in ASI) (this work; [23]), while they share some modulatory functions in ASJ neurons.

**Table 1:** Percent dauer formation at 15°C of *trx-1; daf-c* double and triple mutants.

	*trx-1(+)*		*trx-1(ok1449)*	
Genotype	% ± SEM†	N	% ± SEM†	N
wild type	0±0	478	0±0	442
*daf-11(ks67)* **	0±0	357	92±2	300
*daf-11(m47)* **	8±3	586	98±1	333
*daf-11(sa195)* *	31±4	412	59±8	172
*tax-4(p678)*	2±1	420	0±0	511
*daf-7(e1372)* **	63±4	1257	95±1	754
*daf-1(e1287)* **	13±3	451	51±6	485
*daf-8(e1393)* **	21±3	1826	44±2	1281
*daf-2(e1370)*	0±0	388	0±0	333
*daf-2(e1368)*	0±0	280	0±0	167
*daf-2(m577)*	0±0	250	0±0	187
*pdk-1(sa680)*	37±4	863	44±4	627
*daf-28(tm2308)* **	26±3	617	0±0	578
*daf-28(sa191)*	39±4	318	26±8	238
*daf-2(e1370); daf-28(sa191)* §	na	na	38±1	365

† Mean percentage of dauer larvae ± standard error of the mean (SEM); 3 assays per genotype, except for wild type, *tax-4(p678)*, *daf-28(tm2308), daf-2(e1368), daf-2(m577)* and the triple mutant: 2 assays. N: total (pooled) number of animals.

**p<0.01;

*p<0.05 by chi-squared test.

§ *p*<0.05 by chi-squared test with respect to *trx-1(ok1449); daf-28(sa191)*. na: not assayed.

**Table 2:** Percent dauer formation at 25°C of *trx-1; daf-c* double and triple mutants.

	*trx-1(+)*		*trx-1(ok1449)*	
Genotype	% ± SEM†	N	% ± SEM†	N
wild type	0±0	659	0±0	661
*daf-11(ks67)* **	31±5	547	99±1	422
*daf-11(m47)* **	78±5	421	100±0	474
*daf-11(sa195)* **	97±1	595	82±2	221
*tax-4(p678)*	2±1	853	2±0	931
*daf-2(e1370)*	96±2	435	96±0	466
*daf-2(e1368)*	92±1	356	89±1	349
*daf-2(m577)*	100±0	621	100±0	501
*pdk-1(sa680)*	73±3	980	76±1	1196
*daf-28(tm2308)* **	18±2	781	2±0	570
*daf-28(sa191)* **	85±6	445	18±5	427
*daf-2(e1370); daf-28(sa191)* §§	na	na	96±3	368

† Mean percentage of dauer larvae ± standard error of the mean (SEM); 3 assays per genotype, except for wild type, *tax-4(p678)*, *daf-2(e1368)* and the triple mutant: 2 assays. N: total (pooled) number of animals.

**p<0.01 by chi-squared test.

§§ *p*<0.01 by chi-squared test with respect to *trx-1(ok1449); daf-28(sa191)*. na: not assayed.

**Table 3:** Percent dauer formation at 25°C of *trx-1(ok1449); daf-28(sa191)* double mutants expressing the indicated transgene.

		Transgenic animals		Non-transgenic animals	
Transgene	Transgenic line	% ± SEM†	N	% ± SEM†	N
*Ptrx-1::trx-1::GFP* ++	1	80±1	284	39±3	397
	2	82±3	245	48±7	395
	3	66±2	252	23±1	363
*Pssu-1::trx-1::GFP* ++	1	74±4	441	43±14	471
	2	78±1	514	46±3	496
*Ptrx-1::trx-1(SGPS)::GFP* ++	1	81±3	271	35±6	305
	2	86±3	316	41±1	385
	3	66±6	226	21±5	376
*Pgpa-4::trx-1::GFP*	1	53±14	424	34±9	531
	2	36±10	502	38±9	472
	3	34±9	450	28±7	616
*Pges-1::trx-1::GFP*	1	40±12	518	37±6	503
	2	46±13	501	39±6	507
*Ptrx-1::GFP*	1	43±8	474	30±12	470
	2	48±7	339	30±10	479
	3	34±8	455	31±9	541

† Mean percentage of dauer larvae ± standard error of the mean (SEM); 2 assays per transgenic line, except for *Ptrx-1::GFP* and *Pgpa-4::trx-1::GFP*: 3 assays. N: total (pooled) number of animals.

++ *p*<0.001 by chi-squared test.

### *trx-1* deletion enhances the Daf-c phenotype of mutations in the TGF-beta signaling pathway

There is evidence that DAF-11 acts upstream of the IS and TGF-beta signaling pathways (Figure S1; reviewed in [25], [26]). To determine if *trx-1* interacts with any of these two genetic pathways for dauer formation, we first scored for dauers in double mutants containing *trx-1(ok1449)* and *daf-c* mutations in the TGF-beta signaling pathway. Previously, strong synergy was identified between *daf-c* mutations in several genes of the IS pathway, including *daf-2* and *daf-28*, and mutations in genes of the parallel TGF-beta signaling pathway, represented here by *daf-7*, *daf-1* and *daf-8* [21], [27]. We observed a marked increase in dauer formation in all three *trx-1;TGF-beta* double mutant combinations tested at 15°C (Figure 1A and Table 1). A straightforward interpretation of this synthetic enhancement observed is that the Daf-c phenotype of mutations in the TGF-beta signaling pathway is TRX-1-independent. Since *TGF-beta* single mutants already form nearly 100% dauers at 25°C ([28]; data not shown), *trx-1;TGF-beta* double mutants did not display any further increase in dauer formation at that temperature (data not shown). We conclude that TRX-1 acts independently of the TGF-beta signaling pathway for dauer formation.

### TRX-1 function is needed for the Daf-c phenotype of *daf-28* mutants

To investigate the interaction of *trx-1* with the IS pathway for dauer formation, we first analyzed double mutants of *trx-1* with *daf-28*. *trx-1* suppressed the Daf-c phenotype of the two *daf-28* alleles tested (Figures 1A and 1B; Tables 1 and 2), regardless of their nature (*sa191* is a dominant-negative allele; *tm2308* is predicted to be null) (Table S1). These results indicate that the suppression is not allele-specific and that the Daf-c phenotype of *daf-28* mutants requires TRX-1 function for dauer formation. Our findings suggest that loss of TRX-1 function likely increases insulin-like signaling upon mutation of *daf-28*.

### Suppression of the *daf-28* Daf-c phenotype by *trx-1(ok1449)* depends on DAF-2 insulin-like receptor signaling

To test whether the ability of *trx-1(ok1449)* to suppress the Daf-c phenotype of *daf-28(sa191)* depends on functional DAF-2, we first analyzed dauer formation in double mutants containing the *trx-1(ok1449)* deletion and hypomorphic (partial loss-of-function) mutant alleles of *daf-2* or *pdk-1*. The latter encodes a homologue of the mammalian Akt/PKB kinase PDK1, involved in transducing DAF-2 signals on to the DAF-16 FOXO transcription factor to regulate dauer formation (Figure S1; [29]). Deletion mutations of *daf-16* and *daf-18* (the latter encoding a homologue of the mammalian PTEN tumor suppressor), which act downstream of *daf-2* for dauer formation, suppress the Daf-c phenotype of *daf-2* mutants [27], [30], [31]. In addition, RNA interference of the gene *pptr-1* (encoding a homologue of a B56 regulatory subunit of the mammalian PP2A holoenzyme), which acts downstream of both *daf-2* and *pdk-1* for dauer formation, suppresses the Daf-c phenotypes of both *daf-2* and *pdk-1* mutants [32]. Thus, if TRX-1 acted downstream of DAF-2 and PDK-1, we would expect a suppressive effect of *trx-1(ok1449)* on the Daf-c phenotypes of either *daf-2* or *pdk-1* mutants. However, *trx-1* deletion did not suppress the Daf-c phenotypes of three different hypomorphic *daf-2* mutants or of one *pdk-1* mutant at 15 and 25°C (Figures 1A and 1B; Tables 1 and 2). Moreover, at the intermediate temperature of 20°C, *trx-1* deletion did not suppress the Daf-c phenotype of *daf-2(e1370)* animals: the percent dauer formation ± standard error of the mean was 26±4 for *daf-2(e1370)* and 21±1 for *trx-1(ok1449); daf-2(e1370)*, with n>300 animals in total per genotype (*p*>0.1 by chi-squared test; 2 assays). Together, these data suggest that TRX-1 functions upstream of or in parallel to both DAF-2 and PDK-1 for dauer formation. To investigate whether the suppression of the *daf-28* Daf-c phenotype by *trx-1(ok1449)* depends on DAF-2 signaling, we constructed the triple mutant *trx-1(ok1449); daf-2(e1370); daf-28(sa191)*. If the suppression of *daf-28(sa191)* by *trx-1(ok1449)* was independent of DAF-2 signaling, we would expect the triple mutant to form dauers at levels comparable with those of *trx-1(ok1449); daf-28(sa191)* double mutant animals, indicating that the suppression still persists even after mutation of *daf-2*. However, *trx-1(ok1449); daf-2(e1370); daf-28(sa191)* triple mutant animals formed dauers at levels that were similar to those of *daf-28(sa191)* single mutant animals, at both 15 and 25°C (Figure 1A and 1B; Table 1 and 2), suggesting that the suppression is no longer effective when *daf-2* is mutated. Therefore, although genetic interactions using non-null mutants (cf. Table S1) need to be interpreted with some caution, these data demonstrate that the ability of *trx-1(ok1449)* to suppress the Daf-c phenotype of *daf-28(sa191)* depends on functional DAF-2.

### Redox-independent functions of transgenic TRX-1 specifically in ASJ neurons restore the suppression of *daf-28(sa191)* by *trx-1(ok1449)*

To investigate whether wild-type TRX-1 can restore the suppression of the *daf-28(sa191)* Daf-c phenotype caused by *trx-1* deletion at 25°C, the transgene *Ptrx-1::trx-1::GFP* (see Materials and Methods) was used to transform *trx-1(ok1449); daf-28(sa191)* animals. Transgenic lines expressing this transgene restored the Daf-c phenotype back to *daf-28(sa191)* single mutant levels (Figure 1C and Table 3). Since the GFP-tagged transgene can be visualized in ASJ, this is in agreement with the hypothesis that wild-type *trx-1* acts in ASJ neurons to modulate DAF-28 signaling during dauer formation. To confirm this notion, the expression of wild-type *trx-1* genomic DNA fused to GFP was driven from another ASJ neuron-specific promoter, *ssu-1* [33]. Consistent with our hypothesis, this transgene *Pssu-1::trx-1::GFP* also rescued the suppression of the Daf-c phenotype of *daf-28(sa191)* by *trx-1(ok1449)* (Figure 1C and Table 3).

*C. elegans* TRX-1 shares many conserved residues with its thioredoxin orthologues throughout evolution [9], including the active site, which has extensively been demonstrated to be essential for its oxidoreductase activity in bacteria [5], yeast [34], fruit fly [35], and in humans [36]. To investigate whether the redox activity of TRX-1 is necessary for rescue of *trx-1(ok1449); daf-28(sa191)* double mutants, we transformed these animals with *Ptrx-1::trx-1(SGPS)::GFP,* a transgene containing the mutated active site SGPS instead of the wild-type CGPC. The conversion of thioredoxin active-site cysteines to serines has been widely shown to eliminate its ability to function as oxidoreductase in classical reduction assays [5], [36], [37]. Interestingly, transgenic animals expressing the mutated transgene *Ptrx-1::trx-1(SGPS)::GFP* restored the Daf-c phenotype just as *Ptrx-1::trx-1::GFP* did (Figure 1C and Table 3), indicating that TRX-1 function in dauer formation does not require its oxidoreductase activity.

In contrast, the Daf-c restoration rescue could not be achieved when *trx-1(ok1449); daf-28(sa191)* double mutants either expressed a transgene containing the ASI neuron-specific *gpa-4* promoter [38] with the complete coding region of wild-type *trx-1* fused to GFP (*Pgpa-4::trx-1::GFP*), a transgene that drives expression of wild-type *trx-1::GFP* from the *ges-1* promoter in the intestine (*Pges-1::trx-1::GFP*) [39] or a transgene that only drives GFP expression from the *trx-1* promoter in ASJ neurons (*Ptrx-1::GFP*) (Figure 1C and Table 3). Together, these findings suggest that redox-independent functions of TRX-1 specifically in ASJ neurons are required for the Daf-c phenotype caused by defects in *daf-28* function.

### TRX-1 contributes to the down-regulation of *daf-28* expression during dauer formation, a process likely controlled by DAF-28-mediated feedback regulation

**Figure 2:**
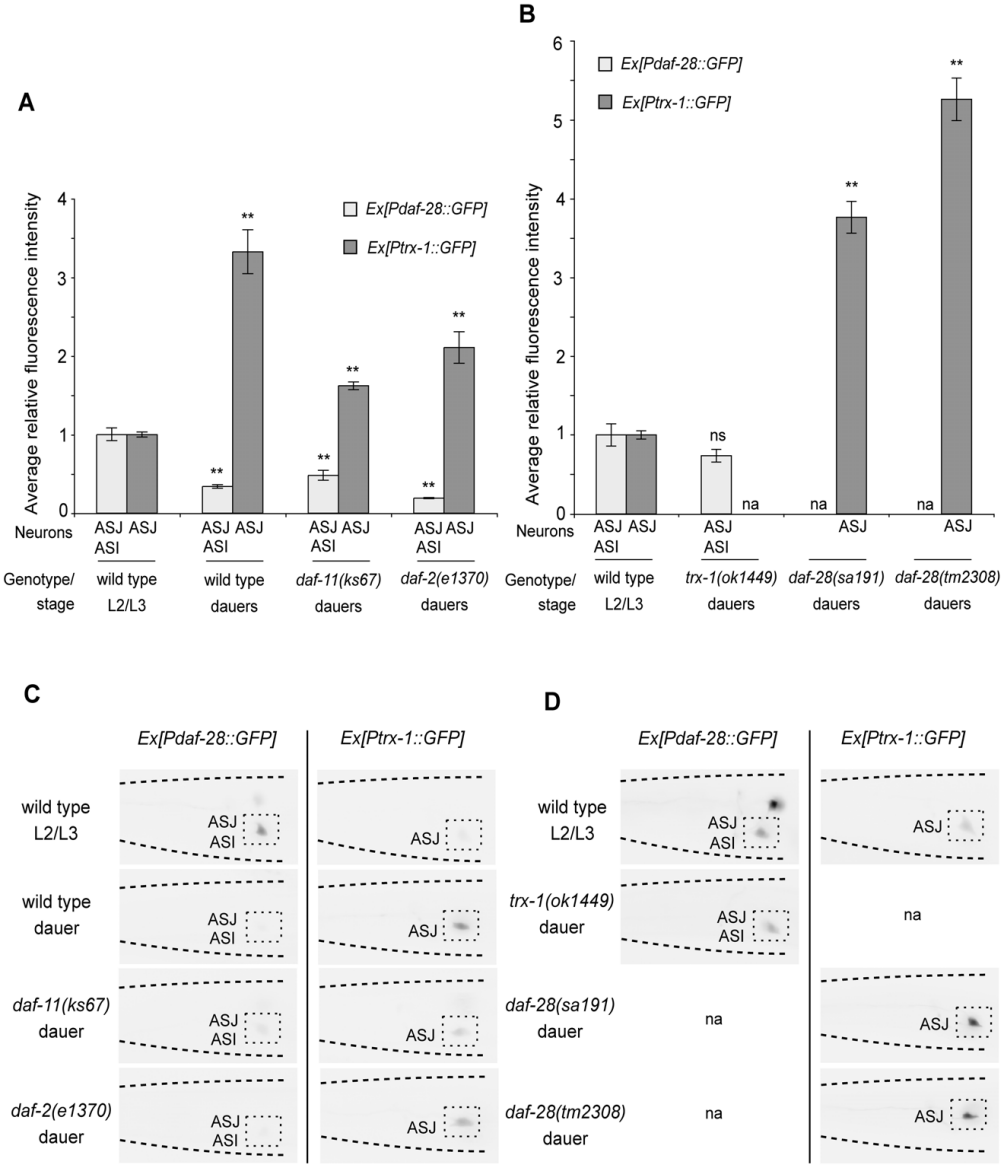
TRX-1 contributes to the down-regulation of *daf-28* insulin-like gene expression during dauer formation. Quantification of *Ptrx-1::GFP* and *Pdaf-28::GFP* expression in dauers and well-fed, growing L2/L3 larvae in ASJ or ASI neurons. Average fluorescence intensity in ASJ or ASI neurons, normalized to that of well-fed, growing L2/L3 wild-type larvae, is shown for wild-type dauers and for *daf-2* and *daf-11* mutant dauers in (A), and for *trx-1* and *daf-28* mutant dauers in (B). Two independent transgenic lines were examined for each of the two transcriptional *Ptrx-1::GFP* and *Pdaf-28::GFP* reporters, and the results were very similar; one transgenic line was tested for *daf-28(tm2308)* dauers. The data derived from one transgenic line are presented. Each bar represents the average relative fluorescence intensity of 28–34 animals ± standard error of the mean (SEM). ** *p*<0.01 relative to L2/L3 wild-type larvae, by Student's *t* test (two-tailed, two-sample unequal variance); na: not assayed; ns: not significant. Shown in (C) and (D) are representative color-inverted images of animals assayed in (A) and (B), respectively, revealing the differences in GFP intensity in ASJ or ASI neurons (boxed). The tip of the head points to the left in all panels.

**Figure 3:**
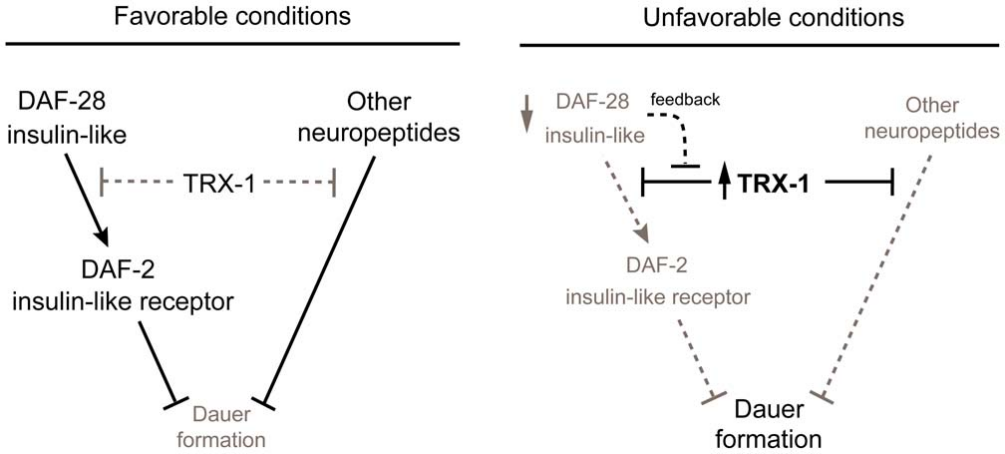
Model for TRX-1 function during dauer formation. TRX-1 is a novel modulator of the insulin-like neuropeptide DAF-28 during dauer formation. It modulates DAF-28 signaling, upstream of DAF-2, by specifically contributing to the down-regulation of *daf-28* expression during dauer formation; this modulatory process is likely subject to DAF-28-mediated feedback regulation (black dashed line). In addition, TRX-1 possibly affects dauer formation independently of both DAF-28 and DAF-2 by modulating other neuropeptides co-expressed in ASJ neurons (see Results and Discussion for details).

Previously, it was shown that *Pdaf-28::GFP* expression in dauers is down-regulated by conditions of starvation and exposure to dauer pheromone [18]. To test whether *trx-1* expression responds to these dauer-promoting signals, we analyzed *Ptrx-1::GFP* expression in both wild-type natural dauers, which are induced by a combination of both signals, and in *daf-c* mutant dauers, represented here by *daf-11* and *daf-2*. Since *trx-1* suppresses the Daf-c phenotype of *daf-28* mutants (cf. above), one could expect that the response of *Ptrx-1::GFP* levels to dauer-promoting signals might be opposite to that of *Pdaf-28::GFP*. Consistent with our hypothesis, *Pdaf-28::GFP* was down-regulated and *Ptrx-1::GFP* was up-regulated in wild-type natural dauers, and in *daf-11* and *daf-2* mutant dauers (Figures 2A and 2C). In contrast, these opposing expression levels observed in dauers were not seen in well-fed, growing L2/L3 larvae of the corresponding genotypes (Figure S2), suggesting a possible regulatory effect of TRX-1 on DAF-28 (or vice versa) only during dauer formation. Therefore, to test whether TRX-1 function contributes to the down-regulation of *daf-28* expression during dauer formation, we analyzed *Pdaf-28::GFP* expression in *trx-1* mutant dauers. If the down-regulation of *daf-28* expression during dauer formation was independent of TRX-1, we would expect *trx-1* mutant dauers to express *Pdaf-28::GFP* at levels comparable with those of wild-type natural dauers (cf. Figure 2A), indicating that the down-regulation still persists even after loss of *trx-1*. Interestingly, we observed that *Pdaf-28::GFP* expression in *trx-1* mutant dauers is not down-regulated, but is in fact not significantly different from what is seen in well-fed, growing L2/L3 wild-type larvae (Figures 2B and 2D). These findings suggest that the up-regulation of TRX-1 specifically during dauer formation is, at least in part, causative of a reduction in DAF-28 signaling. Moreover, the up-regulation of *Ptrx-1::GFP* expression in *daf-28* mutant dauers (Figures 2B and 2D), is further increased when compared with that seen in wild-type natural dauers (cf. Figures 2A and 2C). This result suggests that *trx-1* up-regulation during dauer formation is likely subject to DAF-28-mediated feedback regulation. Together, these results are consistent with a model (Figure 3) in which TRX-1 modulates DAF-28 signaling by contributing to the down-regulation of *daf-28* expression exclusively during dauer formation, a modulatory process that is likely controlled by DAF-28-dependent feedback regulation.

## Discussion

Here, we have identified the thioredoxin TRX-1 as a novel modulator of the insulin-like neuropeptide DAF-28 during dauer formation in *C. elegans*. We found that *trx-1* suppresses the Daf-c phenotypes of all *daf-28* insulin-like mutant alleles tested, and that this suppression is dependent on a functional DAF-2 insulin-like receptor (Figures 1A and 1B; Tables 1 and 2). Genetic rescue experiments showed that redox-independent functions of TRX-1 specifically in ASJ neurons are needed for the Daf-c phenotype caused by defects in *daf-28* function (Figure 1C and Table 3). The suppression observed at the genetic level phenocopied that seen at the cellular level specifically during dauer formation: *Ptrx-1::GFP* and *Pdaf-28::GFP* showed opposing expression patterns in dauers (Figures 2A and 2C), which were not observed in growing L2/L3 larvae (Figure S2). In addition, *Pdaf-28::GFP* down-regulation during dauer formation required functional TRX-1, a mechanism that is likely controlled by DAF-28-dependent feedback regulation (Figures 2B and 2D). Taken together, our results suggest a model in which TRX-1 contributes to the adjustment of *daf-28* insulin-like expression, and consequently its function, during dauer formation (Figure 3).

*C. elegans* neuropeptides are classified into three classes: the insulin-like proteins, the FMRF (Phe-Met-Arg-Phe)-amide peptides (referred to as FLPs) and the neuropeptide-like proteins or NLPs (reviewed in [40]). In addition to DAF-28, it might be possible that TRX-1 also modulates other neuropeptides co-expressed in ASJ neurons (Figure 3), such as the insulin-like proteins INS-9 and INS-1, or NLP-3 and FLP-21 ([24], [41]; reviewed in [40]). If the effect of TRX-1 on dauer formation was solely dependent on DAF-28 signaling, we would expect that overexpression of *trx-1* in the likely null mutant *daf-28(tm2308)* results in no enhancement of the Daf-c phenotype. Interestingly, the observation that overexpression of wild-type *trx-1::GFP* in ASJ was sufficient to enhance the weak Daf-c phenotype of the likely null mutant *daf-28(tm2308)* (Table S2), suggests that TRX-1 in part affects dauer formation independently of DAF-28, and potentially independently of DAF-2 (Figure 3). Moreover, the fact that *trx-1* deletion, like *daf-28(sa191)* [21], enhanced the Daf-c phenotype of TGF-beta pathway mutations and of most *daf-11* mutations, whereas *trx-1* deletion suppressed the Daf-c phenotype of *daf-28* mutations (Figures 1A and 1B; Tables 1 and 2), also supports the notion that TRX-1 to some extent modifies dauer formation independently of DAF-28, and possibly also of DAF-2 (Figure 3). Furthermore, because wild-type animals overexpressing wild-type *trx-1::GFP* in ASJ were not induced to form dauers at 25°C (Table S2), other pathways and/or neurons might act in parallel to contribute, together with TRX-1, to the modulation of DAF-28 signaling. In fact, the cGMP pathway has previously been suggested to regulate DAF-28 signaling during dauer formation (Figure S1; [18]).

ASJ neurons have been shown to regulate dauer formation in concert with other sensory neurons [11], [12], despite their discrete synaptic connectivity (ASK is the only sensory neuron in synaptic contact with ASJ; www.wormatlas.org). The localized function of TRX-1 in ASJ neurons for dauer formation (Figure 1C; Table 3) and the effect of TRX-1 on *daf-28* expression (Figures 2B and 2D) support a model wherein TRX-1 modulates (the levels of) neurosecretory signals emanating from ASJ neurons to regulate dauer formation in a cell non-autonomous fashion. Although our results do not exclude that TRX-1 affects other neurons through direct synaptic connections, the control it exerts on neuropeptidergic signaling would allow TRX-1 to function locally in ASJ neurons and affect dauer formation remotely. Such a model would explain the observation that deletion of *trx-1* modifies the Daf-c phenotypes of mutations in genes not expressed in ASJ neurons (Figure 1A; Table 1) (e.g. *daf-7* is only expressed in ASI neurons) [42]. Similarly, TRX-1 could contribute to the down-regulation of *daf-28* expression in ASI, either through ASJ-derived DAF-28 or other TRX-1-modified neuropeptides released from ASJ neurons.

Previously, mammalian Trx1 has been proposed to participate in the redox regulation that mediates insulin secretion via NADPH as a signaling molecule [43]. However, we have shown in this report that TRX-1 function in dauer formation does not require its redox activity (Figure 1C and Table 3), suggesting that TRX-1 contributes to the down-regulation of *daf-28* expression during dauer formation via mechanisms other than redox regulation of neuropeptide production and/or release.

It has recently been found in mammals that key neuronal lipid metabolites act as hypothalamic signaling mediators that monitor energy status and contribute to maintaining organism metabolic homeostasis (reviewed in [44]). We propose that the thioredoxin TRX-1 might similarly act as a neuronal modulator in ASJ neurons to monitor metabolic status and thus adjust neuropeptide expression, including the insulin-like neuropeptide DAF-28, during dauer formation in response to adverse conditions (Figure 3). In this context, ASJ neurons would monitor the choice between reproductive development and dauer formation by responding to intra-neuronal fluctuations of TRX-1 (Figure 3). In turn, these TRX-1 fluctuations would be triggered in response to signals from the environment and peripheral tissues that reflect the animal's global energy status. Interestingly, the *C. elegans* ASI neurons have been suggested to mediate responses to nutrient availability through neuroendocrine signals that promote adult longevity by sensing similar energy-monitoring molecules within them [45]. Moreover, ASJ neurons have been proposed to influence dauer formation through sulfated neuroendocrine signals [33]. Thus, together with previous observations by others, our findings anticipate a neuroendocrine network in the nematode involved in adjusting the animal's global energy status in response to a changing environment, both during development and adulthood. Future work is needed to identify and dissect the components of such a neuroendocrine network that, including TRX-1, are involved in regulating mechanisms designed to monitor energy homeostasis in varying environmental conditions in the nematode, and most likely also in higher organisms.

## Materials and Methods

### Nematode strains and culture conditions

The standard methods used for culturing *C. elegans* were described previously ([46]; reviewed in [47]). Strains and transgenes used in this work are summarized in Table S3. All strains were grown at 20°C, except for *daf-c* mutant strains, which were grown at 15°C.

### Transgene injection constructs and germline transformation

The *Ptrx-1::trx-1::GFP* translational fusion construct (cf. Figure 1C; Tables 3 and S2) and the *Pdaf-28::GFP* transcriptional fusion construct (cf. Figures 2 and S2) were previously reported [9], [48]. For the *Ptrx-1::GFP* transcriptional fusion construct (cf. Figures 1C, 2 and S2; Table 3), ∼1 kb of sequence upstream of the *trx-1b* splice variant, plus the first 65 nucleotides of the coding sequence were amplified by PCR and cloned in-frame with GFP into the pPD95.77 vector. The *Ptrx-1::trx-1(SGPS)::GFP* translational fusion construct (cf. Figure 1C and Table 3) was obtained by site-directed mutagenesis of the *trx-1* active site, as specified in the QuikChange II Site-Directed Mutagenesis Kit (Stratagene), using the *Ptrx-1::trx-1::GFP* translational fusion construct as a template. The *Pssu-1::trx-1::GFP*, *Pgpa-4::trx-1::GFP* and *Pges-1::trx-1::GFP* translational fusion constructs (cf. Figure 1C and Table 3) included ∼0.5 kb (*ssu-1)* or ∼2.5 kb (*gpa-4* and *ges-1*) of promoter sequence upstream of the respective start codon fused to the full-length *trx-1* genomic DNA fragment, in-frame with GFP, into the pPD95.77 vector. For the experiments shown in Figures 2 and S2, 80 ng/µl of *Ptrx-1::GFP* or 50 ng/µl of *Pdaf-28::GFP* were coinjected with 20 ng/µl of the injection marker *Pelt-2::mCherry* (a gift from Gert Jansen, Rotterdam), into *trx-1(ok1449); daf-28(sa191)* or *trx-1(ok1449)* animals. These extrachromosomal arrays were then crossed into wild-type, *daf-11(ks67)*, *daf-2(e1370)*, *daf-28(sa191)* or *daf-28(tm2308)* animals. For the rescue experiments shown in Figure 1C and Table 3, 40 ng/µl of *Ptrx-1::trx-1::GFP*, *Pssu-1::trx-1::GFP*, *Ptrx-1::trx-1(SGPS)::GFP*, *Pgpa-4::trx-1::GFP*, *Pges-1::trx-1::GFP* or *Ptrx-1::GFP* were coinjected with 30 ng/µl of the injection marker *Punc-122::DsRed* [49], into *trx-1(ok1449); daf-28(sa191)* animals. For the overexpression experiments shown in Table S2, 100 ng/µl of *Ptrx-1::trx-1::GFP* were coinjected with 30 ng/µl of the injection marker *Punc-122::DsRed* into wild-type or *daf-28(tm2308)* animals. Germline transformation was performed as described elsewhere [50].

### Construction of double and triple mutants

To construct *trx-1; daf-c* double mutants, *trx-1* homozygous males were crossed to the appropriate *daf-c* mutant, and F1 cross progeny hermaphrodites were grown singly at 25°C. We singled F2 dauers and allowed them to recover at 15°C. Recovered dauers were considered to be homozygous for the *daf-c* mutation. To construct the *trx-1; tax-4* double mutant, *trx-1; daf-2* homozygous hermaphrodites were crossed to *tax-4* homozygous males. F2 progeny were grown singly at 25°C from F1 cross progeny. *tax-4* homozygous candidates were selected further from F2s that did not segregate dauers at 25°C. To confirm the presence of the *tax-4* mutation, we performed a soluble compound chemotaxis assay, as described elsewhere [51]. To construct the triple mutant *trx-1(ok1449); daf-2(e1370); daf-28(sa191)*, we took advantage of the fact that *daf-28* dauers recover well at 25°C, whereas *daf-2* dauers do not recover or only recover with difficulties at this temperature [21], [52]. In brief, *trx-1* homozygous males where crossed to *trx-1; daf-2* double mutant hermaphrodites. The resulting male progeny were crossed to *trx-1; daf-28* hermaphrodites, and F1 cross progeny hermaphrodites were grown individually at 25°C. We then picked many F2 dauers from several F1s onto new, seeded plates and kept them at 25°C. Those dauers that recovered after ∼14–24 h, were considered to be *daf-28* homozygotes. F3 progeny of these *daf-28* homozygous candidates that remained arrested as dauers after ∼17–24 h at 25°C were potential *daf-2* homozygotes. We singled these dauers and transferred them to 15°C for recovery. We tested all candidates for the presence of the *daf-28* and *daf-2* mutations by sequencing. In all cases, the presence of the *trx-1* deletion was demonstrated by performing PCR on single-worm lysates, based on methods previously described ([53]; reviewed in [54]).

### Analysis of dauer formation

For dauer formation assays, 8–20 gravid hermaphrodites were allowed to lay eggs for a given time at the test temperature (Tables S4 and S5), and then removed. Dauers and non-dauers (L3 and L4 larvae and adult animals) on the agar and side of the plate were then counted at specific time points after egg-laying ended (Table S4). The scoring time points were selected for each genotype so that all animals had passed the pre-dauer or L2 stages at the time of scoring. Corresponding single and double mutant strains were always assayed in parallel. In all cases, dauers were discriminated from non-dauers based on the absence of pharyngeal pumping, intestinal re-organization and radial shrinkage of the body [13]. More than 165 animals were counted in a total of 2–3 independent assays per genotype. Dauers of selected key genotypes used in this study were subjected to morphological analysis using differential interference contrast (DIC) microscopy at an optical magnification of x800 or x1000, and also tested for resistance to 1% sodium dodecylsulfate (SDS) [13]. All dauers analyzed were identical to wild-type dauers in their morphological features (i.e. pharynx and alae) and SDS-resistance.

### Microscopy and fluorescence imaging

The average relative intensity of a transcriptional *Ptrx-1::GFP* or *Pdaf-28::GFP* reporter was assessed by quantifying GFP intensity in ASJ neurons of dauers or growing L2/L3 larvae mutant for a specific *daf-c* gene as compared to growing L2/L3 wild-type larvae. Animals were visualized on a Zeiss Axioplan fluorescence microscope at an optical magnification of x640. Worms were put into M9 buffer on a very thin 2% agarose pad containing an anesthetic (15 mM NaN_3_). Previously, it had been shown that exposure to 20 mM NaN_3_ or less during 60 min [55], ∼30 mM NaN_3_ for 20 min [56] or 50 mM NaN_3_ for 5 min [57] can induce evident physiological changes in the worm. All animals assayed in our study have been exposed to only 15 mM NaN_3_ for a maximum of 10–15 min prior to image acquisition, thereby avoiding the induction of evident physiological changes. A Hamamatsu CCD camera and Openlab software (Improvision) were used for image acquisition at the brightest focal plane and a fixed exposure time. Pixel intensity in the entire ASJ cell body was determined from captured images in the form of maximum gray values by using NIH ImageJ software. An exception was made for *Pdaf28::GFP* expression intensity in dauers, which was measured from either ASJ or ASI, due to the fact that overall very low expression levels hindered correct neuron identification. Since the brightest expressing cell was always measured for every dauer, the data for *Pdaf-28::GFP* expression intensity in dauers, therefore, is necessarily a conservative (over-) estimate. In all cases, fold differences with respect to growing L2/L3 wild-type larvae were calculated to show the average relative intensity among the assayed animals. Wild-type or mutant dauers and growing L2/L3 *daf-c* mutant larvae were always assayed in parallel with growing L2/L3 wild-type larvae at the test temperature. In brief, wild-type, *trx-1(ok1449), daf-28(sa191)* and *daf-28(tm2308)* dauers were collected from crowded starved plates maintained at 20°C, while *daf-11(ks67)* and *daf-2(e1370)* dauers were picked from uncrowded well-fed plates grown at 25°C; growing L2/L3 *daf-c* mutant larvae were collected from uncrowded well-fed plates maintained at 20°C. Growing L2/L3 wild-type larvae were always picked from uncrowded well-fed plates. Twenty-eight to thirty-six animals were analyzed per genotype and condition. Two independent extrachromosomal transgenic lines were examined for each of the two transcriptional *Ptrx-1::GFP* and *Pdaf-28::GFP* reporters, and the results were very similar; one transgenic line was tested for *daf-28(tm2308)* dauers. The data derived from one transgenic line are presented in Figures 2 and S2. To clarify whether the expression of *Pdaf-28::GFP* in ASJ and ASI changed in a similar fashion among dauers of the four genotypes assayed, we focused our attention on dauers showing expression in at least three of the four possible neuronal cell bodies in the head (ASJL/R, ASIL/R). This approach seeks to exclude expression changes that are due to transgenic extrachromosomal array variability or to transgene mosaicism. The expression of *Pdaf-28::GFP* changed in a similar manner in all cell bodies examined (n = 80 dauers; data not shown), suggesting that similar changes of *Pdaf-28::GFP* expression occur in ASJ and ASI.

## Supporting Information

Figure S1 **A speculative model describing the genetic pathways that regulate dauer formation.** Not all genes known to act in these pathways are shown. Solid lines represent active regulation, and dashed lines represent inactive regulation. Arrows indicate positive regulation and crossbars indicate repressive regulation. See text for details and references.
(TIF)

Figure S2 ***Ptrx-1::GFP*** **levels are similar to** ***Pdaf-28::GFP*** **levels in growing L2/L3 larvae.** The opposing expression levels observed in dauers (cf. Figures 2A and 2C) were not seen in growing L2/L3 larvae. Average fluorescence intensity in ASJ or ASI neurons, normalized to that of growing L2/L3 wild-type larvae, is shown for growing L2/L3 larvae mutant for the indicated *daf-c* genes. Two independent transgenic lines were examined for each of the two transcriptional *Ptrx-1::GFP* and *Pdaf-28::GFP* reporters, and the results were very similar. The data derived from one transgenic line are presented. Each bar represents the average relative fluorescence intensity of 28–36 animals ± standard error of the mean (SEM).
(TIF)

Table S1 **Molecular identities of all mutant alleles used in this study.**
(DOC)

Table S2 **Percent dauer formation at 25°C of animals overexpressing the extrachromosomal array transgene** ***Ptrx-1::trx-1::GFP*** **at 100 ng/µl.**
(DOC)

Table S3 **Strains and extrachromosomal arrays used in this study.**
(DOC)

Table S4 **Egg-laying periods and post-egglay scoring time points for the analysis of dauer formation.**
(DOC)

Table S5 **Percent dauer recovery at 15°C of the egg-laying defective (** ***egl*** **) mutants used in this study.**
(DOC)
