# Supplementary figures and images for: The Thioredoxin TRX-1 Modulates the Function of the Insulin-Like Neuropeptide DAF-28 during Dauer Formation in Caenorhabditis elegans
Source: PLoS One. 2011 Jan 27;6(1):e16561. doi: 10.1371/journal.pone.0016561 (PMC3029385; doi:10.1371/journal.pone.0016561)

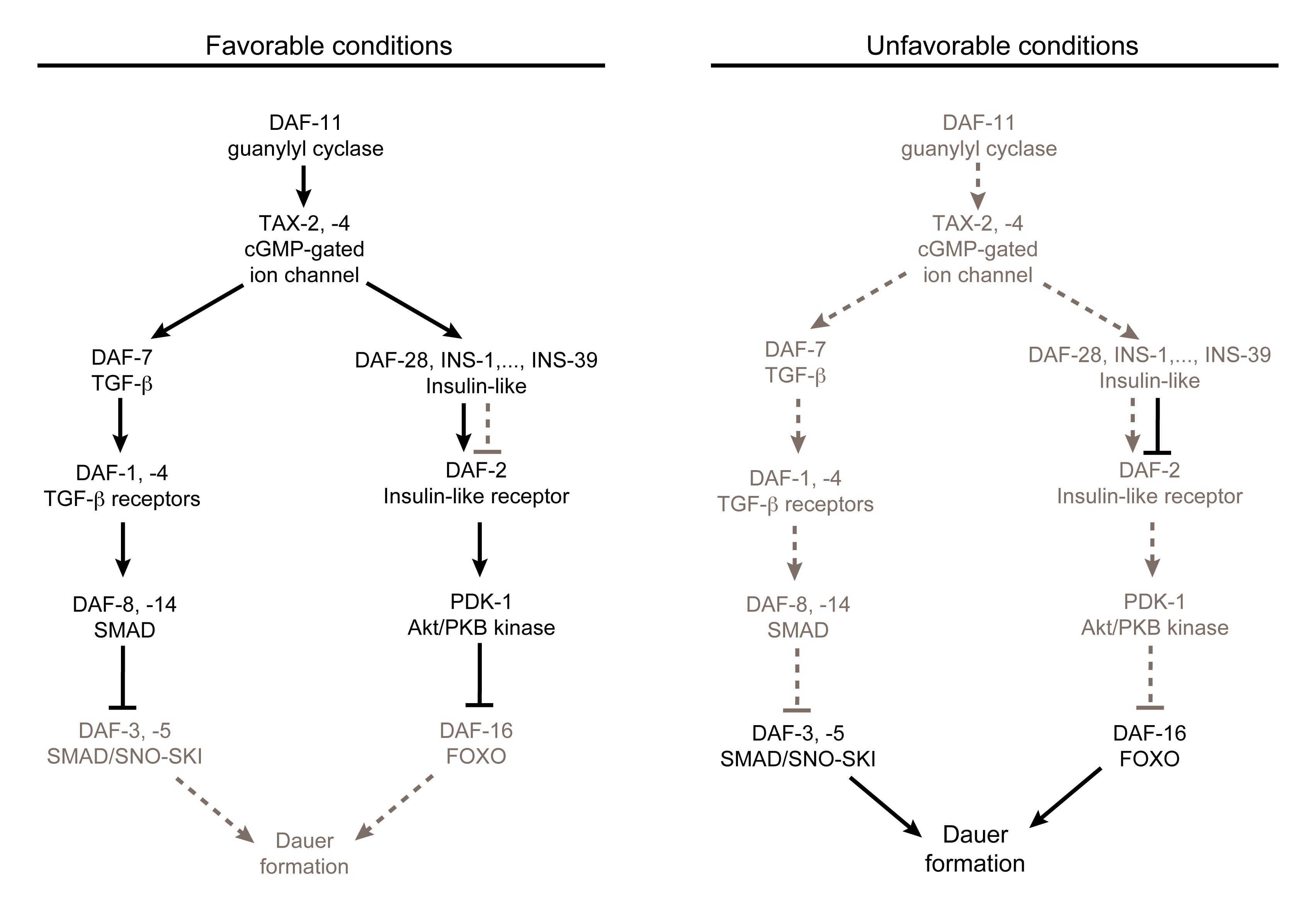

Supplement: Figure S1 — A speculative model describing the genetic pathways that regulate dauer formation. Not all genes known to act in these pathways are shown. Solid lines represent active regulation, and dashed lines represent inactive regulation. Arrows indicate positive regulation and crossbars indicate repressive regulation. See text for details and references. (TIF) [file pone.0016561.s001.tif]

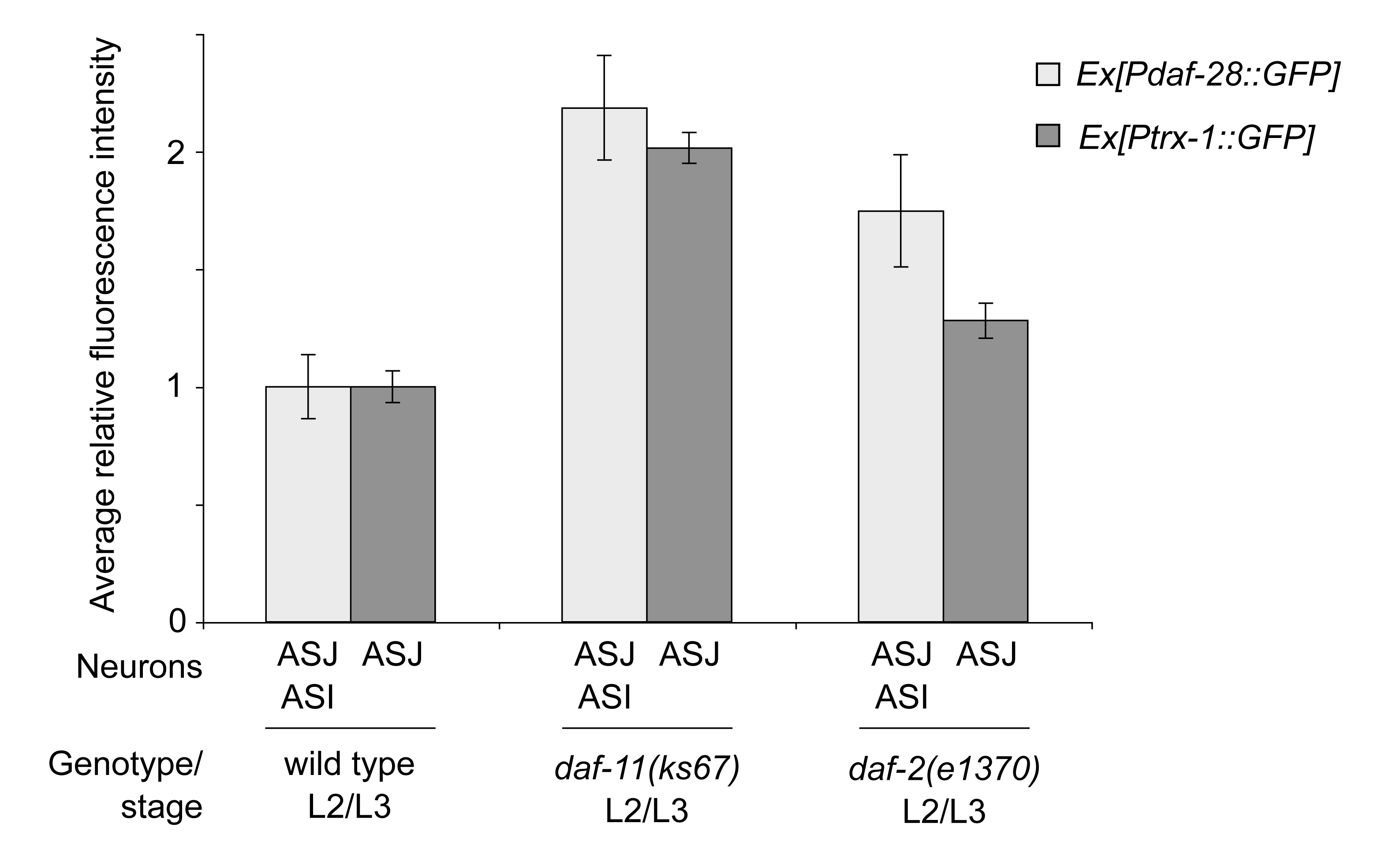

Supplement: Figure S2 — Ptrx-1::GFP levels are similar to Pdaf-28::GFP levels in growing L2/L3 larvae. The opposing expression levels observed in dauers (cf. Figures 2A and 2C) were not seen in growing L2/L3 larvae. Average fluorescence intensity in ASJ or ASI neurons, normalized to that of growing L2/L3 wild-type larvae, is shown for growing L2/L3 larvae mutant for the indicated daf-c genes. Two independent transgenic lines were examined for each of the two transcriptional Ptrx-1::GFP and Pdaf-28::GFP reporters, and the results were very similar. The data derived from one transgenic line are presented. Each bar represents the average relative fluorescence intensity of 28–36 animals ± standard error of the mean (SEM). (TIF) [file pone.0016561.s002.tif]
