# Supplementary material for: The Thioredoxin TRX-1 Modulates the Function of the Insulin-Like Neuropeptide DAF-28 during Dauer Formation in Caenorhabditis elegans
Source: PLoS One. 2011 Jan 27;6(1):e16561. doi: 10.1371/journal.pone.0016561 (PMC3029385; doi:10.1371/journal.pone.0016561)
Supplement: Table S1 — Molecular identities of all mutant alleles used in this study. (DOC) [file pone.0016561.s003.doc]

**Table S1.** Molecular identities of all mutant alleles used in this study.

| **Gene** | **Allele** | **Molecular lesion** | **Additional notes** | **References** |
| --- | --- | --- | --- | --- |
| *trx-1* | *ok1449* | 863bp deletion / 4bp insertion | Deletion removes part of the proximal *trx-1* promoter and all but the last exon of the *trx-1* gene: null mutant. | [1] |
| *daf-11* | *ks67* | G-to-A substitution | Missense mutation of D770 to N in the guanylyl cyclase domain; loss-of-function mutation. | [2] |
|  | *m47* | unknown | Nonsense mutation in the predicted kinase domain, leads to deletion of the entire guanylyl cyclase domain; loss-of-function mutation. | [2] |
|  | *sa195* | C-to-T substitution | Nonsense mutation at Q450; predicted to truncate DAF-11 before the kinase domain; best candidate for a *daf-11* molecular null allele. | [3] |
| *tax-4* | *p678* | C-to-T substitution | Nonsense mutation at Q82; predicted to truncate TAX-4 before the first putative transmembrane domain. Null mutant. | [4] |
| *daf-7* | *e1372* | G-to-A substitution | Splice site at exon2/intron2 transition changed from GT to AT; results in a loss-of-function mutation. | http://www.  wormbase.org |
| *daf-1* | *e1287* | G-to-A substitution | Nonsense mutation at W202; results in a loss-of-function mutation. | [5] |
| *daf-8* | *e1393* | C-to-T substitution | Missense mutation of S391 to L in the Mad homology 2 (MH2) domain; loss-of-function mutation. | [6,7] |

**Table S1**. (continued).

| **Gene** | **Allele** | **Molecular lesion** | **Additional notes** | **References** |
| --- | --- | --- | --- | --- |
| *daf-2* | *e1370* | C-to-T substitution | Missense mutation of P1465 to S in the tyrosine kinase domain; results in loss-of-function. Class 2 allele. | [8,9] |
|  | *e1368* | C-to-T substitution | Missense mutation of S573 to L in the ligand binding domain; results in loss-of-function. Class 1 allele. | [8,9] |
|  | *m577* | G-to-A substitution | Missense mutation of C1042 to Y in exon 13 (at tyrosine kinase domain or right before it); results in loss-of-function. Class 1 allele. | [8]  http://www.  wormbase.org |
| *pdk-1* | *sa680* | G-to-A (or C) substitution | Missense mutation of G295 to R in the kinase domain; results in loss-of-function. | [10]  http://www.  wormbase.org |
| *daf-28* | *sa191* | C-to-T substitution | Missense mutation of R37 to C in the probable proteolytic cleavage site. *sa191* is a dominant-negative allele that poisons *daf-28(+)* function. | [11,12] |
|  | *tm2308* | 156bp deletion | Deletion removes part of the 3'-end of the first exon, including the proteolytic cleavage site, and the 5'-end of the only intron, which introduces two in-frame stop codons. Predicted to be null. | http://www.  wormbase.org |

**References**

1. Miranda-Vizuete A, Fierro González JC, Gahmon G, Burghoorn J, Navas P, et al. (2006) Lifespan decrease in a *Caenorhabditis elegans* mutant lacking TRX-1, a thioredoxin expressed in ASJ sensory neurons. FEBS Lett 580: 484-490.

2. Murakami M, Koga M, Ohshima Y (2001) DAF-7/TGF-beta expression required for the normal larval development in *C. elegans* is controlled by a presumed guanylyl cyclase DAF-11. Mech Dev 109: 27-35.

3. Birnby DA, Link EM, Vowels JJ, Tian H, Colacurcio PL, et al. (2000) A transmembrane guanylyl cyclase (DAF-11) and Hsp90 (DAF-21) regulate a common set of chemosensory behaviors in *Caenorhabditis elegans*. Genetics 155: 85-104.

4. Komatsu H, Mori I, Rhee JS, Akaike N, Ohshima Y (1996) Mutations in a cyclic nucleotide-gated channel lead to abnormal thermosensation and chemosensation in *C. elegans*. Neuron 17: 707-718.

5. Gunther CV, Georgi LL, Riddle DL (2000) A *Caenorhabditis elegans* type I TGF beta receptor can function in the absence of type II kinase to promote larval development. Development 127: 3337-3347.

6. Park D, Estevez A, Riddle DL (2010) Antagonistic Smad transcription factors control the dauer/non-dauer switch in *C. elegans*. Development 137: 477-485.

7. Tewari M, Hu PJ, Ahn JS, Ayivi-Guedehoussou N, Vidalain PO, et al. (2004) Systematic interactome mapping and genetic perturbation analysis of a *C. elegans* TGF-beta signaling network. Mol Cell 13: 469-482.

8. Gems D, Sutton AJ, Sundermeyer ML, Albert PS, King KV, et al. (1998) Two pleiotropic classes of *daf-2* mutation affect larval arrest, adult behavior, reproduction and longevity in *Caenorhabditis elegans*. Genetics 150: 129-155.

9. Kimura KD, Tissenbaum HA, Liu Y, Ruvkun G (1997) *daf-2*, an insulin receptor-like gene that regulates longevity and diapause in *Caenorhabditis elegans*. Science 277: 942-946.

10. Paradis S, Ailion M, Toker A, Thomas JH, Ruvkun G (1999) A PDK1 homolog is necessary and sufficient to transduce AGE-1 PI3 kinase signals that regulate diapause in *Caenorhabditis elegans*. Genes Dev 13: 1438-1452.

11. Li W, Kennedy SG, Ruvkun G (2003) *daf-28* encodes a *C. elegans* insulin superfamily member that is regulated by environmental cues and acts in the DAF-2 signaling pathway. Genes Dev 17: 844-858.

12. Malone EA, Thomas JH (1994) A screen for nonconditional dauer-constitutive mutations in *Caenorhabditis elegans*. Genetics 136: 879-886.
