# Supplementary material for: The Thioredoxin TRX-1 Modulates the Function of the Insulin-Like Neuropeptide DAF-28 during Dauer Formation in Caenorhabditis elegans
Source: PLoS One. 2011 Jan 27;6(1):e16561. doi: 10.1371/journal.pone.0016561 (PMC3029385; doi:10.1371/journal.pone.0016561)
Supplement: Table S2 — Percent dauer formation at 25°C of animals overexpressing the extrachromosomal array transgene Ptrx-1::trx-1::GFP at 100 ng/µl. (DOC) [file pone.0016561.s004.doc]

**Table S2. Percent dauer formation at 25°C of animals overexpressing the extrachromosomal array transgene *Ptrx-1::trx-1::GFP*** at 100 ng/µl.

|  |  |  | **Transgenic animals** | | **Non-transgenic animals** | |  |
| --- | --- | --- | --- | --- | --- | --- | --- |
| **Genotype** | **Transgenic line** | **Assay** | **%** | **N** | **%** | **N** | ***p*-value** |
| wild type | 1 | 1 | 0 | 170 | 0 | 260 | - |
|  |  | 2 | 0 | 280 | 0 | 334 | - |
|  | 2 | 1 | 0 | 239 | 0 | 301 | - |
|  |  | 2 | 0 | 294 | 0 | 300 | - |
| *daf-28(tm2308)* | 1 | 1 | 10 | 250 | 3 | 303 | <0.001 |
|  |  | 2 | 5 | 258 | 1 | 343 | 0.003 |
|  | 2 | 1 | 14 | 237 | 1 | 286 | <0.001 |
|  |  | 2 | 3 | 248 | 1 | 315 | 0.19 |

N: total number of animals. The chi-squared test was used to determine *p*-values; less than 0.05 are considered statistically significant. See Materials and Methods for details.
