# Supplementary material for: The Thioredoxin TRX-1 Modulates the Function of the Insulin-Like Neuropeptide DAF-28 during Dauer Formation in Caenorhabditis elegans
Source: PLoS One. 2011 Jan 27;6(1):e16561. doi: 10.1371/journal.pone.0016561 (PMC3029385; doi:10.1371/journal.pone.0016561)
Supplement: Table S3 — Strains and extrachromosomal arrays used in this study. (DOC) [file pone.0016561.s005.doc]

**Table S3. Strains and extrachromosomal arrays used in this study.**

A. Strains

| **Strain** | **Genotype** |
| --- | --- |
| JT5010^§^, JT5009^†^ | wild-type N2 Bristol |
| VZ1^§^, OE3256^†^ | *trx-1(ok1449) II* |
| OE3448 | *daf-11(ks67) V* |
| OE3406 | *daf-11(m47) V* |
| JT195 | *daf-11(sa195) V* |
| PR678 | *tax-4(p678) III* |
| CB1372 | *daf-7(e1372) III* |
| CB1287 | *daf-1(e1287) IV* |
| CB1393 | *daf-8 (e1393) I* |
| JT191 | *daf-28(sa191) V* |
| QZ83 | *daf-28(tm2308) V* |
| CB1370 | *daf-2(e1370) III* |
| DR1572 | *daf-2(e1368) III* |
| DR1567 | *daf-2(m577) III* |
| JT9609 | *pdk-1(sa680) X* |
| OE3550 | *trx-1(ok1449) II; daf-11(ks67) V* |
| OE3551 | *trx-1(ok1449) II; daf-11(m47) V* |
| VZ8 | *trx-1(ok1449) II; daf-11(sa195) V* |
| OE3570 | *trx-1(ok1449) II; tax-4(p678) III* |
| VZ6 | *trx-1(ok1449) II; daf-7(e1372) III* |
| OE3815 | *trx-1(ok1449) II; daf-1(e1287) IV* |
| OE3568 | *daf-8 (e1393) I; trx-1(ok1449) II* |
| OE3293 | *trx-1(ok1449) II; daf-28(sa191) V* |
| OE3572 | *trx-1(ok1449) II; daf-28(tm2308) V* |
| VZ5 | *trx-1(ok1449) II; daf-2(e1370) III* |
| VZ11 | *trx-1(ok1449) II; daf-2(e1368) III* |
| VZ10 | *trx-1(ok1449) II; daf-2(m577) III* |
| OE4060 | *trx-1(ok1449) II; pdk-1(sa680) X* |
| OE4068 | *trx-1(ok1449) II; daf-2(e1370) III; daf-28(sa191) V* |

^§^Hermaphrodites. ^†^Male stock.

**Table S3**. (continued).

| **Strain** | **Genotype** |
| --- | --- |
| OE4055 | *ofEx345* |
| OE4061 | *daf-11(ks67) V; ofEx345* |
| OE4062 | *daf-2(e1370) III; ofEx345* |
| OE3490 | *trx-1(ok1449) II; ofEx345* |
| OE3803 | *ofEx379* |
| OE4056 | *daf-11(ks67) V; ofEx379* |
| OE4057 | *daf-2(e1370) III; ofEx379* |
| OE4058 | *daf-28(sa191) V; ofEx379* |
| OE4080 | *daf-28(tm2308) V; ofEx379* |
| OE4064 | *trx-1(ok1449) II; daf-28(sa191) V; ofEx416* |
| OE4065 | *trx-1(ok1449) II; daf-28(sa191) V; ofEx417* |
| OE4081 | *trx-1(ok1449) II; daf-28(sa191) V; ofEx424* |
| OE4082 | *trx-1(ok1449) II; daf-28(sa191) V; ofEx1050* |
| OE4066 | *trx-1(ok1449) II; daf-28(sa191) V; ofEx418* |
| OE4067 | *trx-1(ok1449) II; daf-28(sa191) V; ofEx419* |
| OE4071 | *trx-1(ok1449) II; daf-28(sa191) V; ofEx422* |
| OE4072 | *trx-1(ok1449) II; daf-28(sa191) V; ofEx423* |
| OE4087 | *trx-1(ok1449) II; daf-28(sa191) V; ofEx1055* |
| OE4088 | *trx-1(ok1449) II; daf-28(sa191) V; ofEx1056* |
| OE4069 | *trx-1(ok1449) II; daf-28(sa191) V; ofEx420* |
| OE4070 | *trx-1(ok1449) II; daf-28(sa191) V; ofEx421* |
| OE4083 | *ofEx1051* |
| OE4084 | *ofEx1052* |
| OE4085 | *daf-28(tm2308) V; ofEx1053* |
| OE4086 | *daf-28(tm2308) V; ofEx1054* |

**Table S3. (continued).**

B. Extrachromosomal arrays.

| **Transgene** | **Genotype** |
| --- | --- |
| *ofEx345* | *[Pdaf-28::GFP Pelt-2::mCherry]* |
| *ofEx379* | *[Ptrx-1::GFP Pelt-2::mCherry]* |
| *ofEx416*, *ofEx417* | *[Ptrx-1::trx-1::GFP Punc-122::DsRed]* |
| *ofEx424, ofEx1050* | *[Pssu-1::trx-1::GFP Punc-122::DsRed]* |
| *ofEx418, ofEx419* | *[Ptrx-1::trx-1(SGPS)::GFP Punc-122::DsRed]* |
| *ofEx422, ofEx423* | *[Pgpa-4::trx-1::GFP Punc-122::DsRed]* |
| *ofEx1055, ofEx1056* | *[Pges-1::trx-1::GFP Punc-122::DsRed]* |
| *ofEx420, ofEx421* | *[Ptrx-1::GFP Punc-122::DsRed]* |
| *ofEx1051–ofEx1054* | *[Ptrx-1::trx-1::GFP Punc-122::DsRed]* |
