# Supplementary material for: The Thioredoxin TRX-1 Modulates the Function of the Insulin-Like Neuropeptide DAF-28 during Dauer Formation in Caenorhabditis elegans
Source: PLoS One. 2011 Jan 27;6(1):e16561. doi: 10.1371/journal.pone.0016561 (PMC3029385; doi:10.1371/journal.pone.0016561)
Supplement: Table S4 — Egg-laying periods and post-egglay scoring time points for the analysis of dauer formation. (DOC) [file pone.0016561.s006.doc]

**Table S4**. Egg-laying periods and post-egglay scoring time points for the analysis of dauer formation.

|  | **Egg-laying period (h)** | | **Post-egglay scoring time point (h)** | |
| --- | --- | --- | --- | --- |
| **Genotype** | **15°C** | **25°C** | **15°C** | **25°C** |
| wild type | 6 | 3 | 96 | 48 |
| *daf-11* | 6 | 4 | 96 | 66 |
| *tax-4* | 5 | 4 | 96 | 49 |
| *daf-7 ** | 12 | 4 | 96 | 69 |
| *daf-1 ** | 16 | 12 | 96 | 69 |
| *daf-8 ** | 17 | na | 100 | na |
| *daf-2* | 3 | 3 | 96 | 69 |
| *pdk-1 ** | 24 | 21 | 98 | 53 |
| *daf-28* ^†§+^ | 5 | 4 | 96 | 50 |

* These mutants were allowed to lay eggs for longer because they are defective in egg-laying (Egl) [1,2]. The *daf-7* single and double mutant with *trx-1(ok1449)* were also assayed using an egg-laying period of 8 h at 15°C, and the results were very similar (cf. Figure 1A and Table 1): the percent dauer formation ± standard error of the mean was 10 ± 1 for *daf-7(e1372)* and 87 ± 9 for *trx-1(ok1449); daf-7(e1372)*, with n > 145 animals in total per genotype. Tight synchrony was not needed because these strains show dauer recovery defects [2,3,4], and dauer recovery of the respective single and double *egl* mutant with *trx-1(ok1449)* at 15°C was very similar (Table S5). Thus, the effect of *trx-1(ok1449)* on the Daf-c phenotype of the respective *egl* mutation (cf. Figures 1A and 1B; Tables 1 and 2) is independent of dauer recovery*.*

^†^ Dauer formation at 25°C was scored 50 h after egg-laying ended because these dauers recover quickly [5,6].

^§^ The same conditions were applied for the rescue experiments shown in Figure 1C and Table 3, and for the overexpression experiments shown in Table S2; non-transgenic progeny segregating from the same transgenic parents were always scored in parallel as a control.

^+^ The triple mutant (cf. Figures 1A and 1B; Tables 1 and 2) was assayed using the same conditions; the respective double mutants *trx-1(ok1449); daf-2(e1370)* and *trx-1(ok1449); daf-28(sa191)* were always assayed in parallel as a control.

na: not assayed.

**References**

1. Trent C, Tsung N, Horvitz HR (1983) Egg-laying defective mutants of the nematode *Caenorhabditis elegans.* Genetics 104**:** 619-647.

2. Paradis S, Ailion M, Toker A, Thomas JH, Ruvkun G (1999) A PDK1 homolog is necessary and sufficient to transduce AGE-1 PI3 kinase signals that regulate diapause in *Caenorhabditis elegans*. Genes Dev 13: 1438-1452.

3. Riddle DL, Swanson MM, Albert PS (1981) Interacting genes in nematode dauer larva formation. Nature 290: 668-671.

4. Ren P, Lim CS, Johnsen R, Albert PS, Pilgrim D, et al. (1996) Control of *C. elegans* larval development by neuronal expression of a TGF-beta homolog. Science 274: 1389-1391.

5. Malone EA, Thomas JH (1994) A screen for nonconditional dauer-constitutive mutations in *Caenorhabditis elegans*. Genetics 136: 879-886.

6. Malone EA, Inoue T, Thomas JH (1996) Genetic analysis of the roles of *daf-28* and *age-1* in regulating *Caenorhabditis elegans* dauer formation. Genetics 143: 1193-1205.
