# Supplementary material for: The Thioredoxin TRX-1 Modulates the Function of the Insulin-Like Neuropeptide DAF-28 during Dauer Formation in Caenorhabditis elegans
Source: PLoS One. 2011 Jan 27;6(1):e16561. doi: 10.1371/journal.pone.0016561 (PMC3029385; doi:10.1371/journal.pone.0016561)
Supplement: Table S5 — Percent dauer recovery at 15°C of the egg-laying defective ( egl ) mutants used in this study. (DOC) [file pone.0016561.s007.doc]

**Table S5. Percent dauer recovery at 15°C of the egg-laying defective (*egl***) mutants used in this study.

|  | ***trx-1(+)*** | | ***trx-1(ok1449)*** | |
| --- | --- | --- | --- | --- |
| **Genotype** | **%** | **N** | **%** | **N** |
| *daf-2(e1370)* | 100 | 40 | 100 | 39 |
| *daf-7(e1372)* | 82 | 34 | 100 | 48 |
| *daf-1(e1287)* | 23 | 22 | 24 | 34 |
| *pdk-1(sa680)** | 0 | 26 | 0 | 35 |

Dauer recovery of the respective single and double *egl* mutant with *trx-1(ok1449)* at 15°C was very similar. Thus, the effect of *trx-1(ok1449)* on the Daf-c phenotype of the respective *egl* mutation (cf. Figures 1A and 1B; Tables 1 and 2) is independent of dauer recovery. To assay for dauer recovery, dauers were induced by starvation. Following dauer formation, dauers were incubated at 15°C on plates seeded with OP50 bacteria. Dauers and recovered worms on the agar were counted 24 h later to measure dauer recovery. Animals that dried on the plastic sides of the plate were censored from the assay. Dauers were scored as recovered worms if they had resumed pharyngeal pumping. *daf-2(e1370)* was used as a non-Egl control. The respective single and double mutants were always assayed in parallel. N: total number of animals assayed. *dauers of *pdk-1(sa680)* single and double mutant with *trx-1(ok1449)* did not recover at 15°C when incubated on food for more than 3 weeks (data not shown; cf. [1]).

**References**

1. Paradis S, Ailion M, Toker A, Thomas JH, Ruvkun G (1999) A PDK1 homolog is necessary and sufficient to transduce AGE-1 PI3 kinase signals that regulate diapause in *Caenorhabditis elegans*. Genes Dev 13: 1438-1452.
